# Chitosan-Based Biomaterial Scaffolds for the Repair of Infected Bone Defects

**DOI:** 10.3389/fbioe.2022.899760

**Published:** 2022-05-04

**Authors:** Yuhang Tian, Danhua Wu, Dankai Wu, Yutao Cui, Guangkai Ren, Yanbing Wang, Jincheng Wang, Chuangang Peng

**Affiliations:** ^1^ Orthopedic Medical Center, The Second Hospital of Jilin University, Changchun, China; ^2^ The People’s Hospital of Chaoyang District, Changchun, China

**Keywords:** chitosan, bone defect, hydrogel, antibacterial, scaffold

## Abstract

The treatment of infected bone defects includes infection control and repair of the bone defect. The development of biomaterials with anti-infection and osteogenic ability provides a promising strategy for the repair of infected bone defects. Owing to its antibacterial properties, chitosan (an emerging natural polymer) has been widely studied in bone tissue engineering. Moreover, it has been shown that chitosan promotes the adhesion and proliferation of osteoblast-related cells, and can serve as an ideal carrier for bone-promoting substances. In this review, the specific molecular mechanisms underlying the antibacterial effects of chitosan and its ability to promote bone repair are discussed. Furthermore, the properties of several kinds of functionalized chitosan are analyzed and compared with those of pure chitosan. The latest research on the combination of chitosan with different types of functionalized materials and biomolecules for the treatment of infected bone defects is also summarized. Finally, the current shortcomings of chitosan-based biomaterials for the treatment of infected bone defects and future research directions are discussed. This review provides a theoretical basis and advanced design strategies for the use of chitosan-based biomaterials in the treatment of infected bone defects.

## 1 Introduction

Bone defects caused by infection pose a great challenge to orthopedic surgeons (Cui et al., 2017). Although bone tissue exhibits self-healing ability, infection with a pathogen may seriously impair the regeneration process (Dreifke et al., 2013). The current clinical treatment of infected bone defects includes infection control and local reconstruction of the defect. Infection control requires surgical debridement of necrotic and infected tissue, followed by extensive treatment with systemic antibiotics. Nevertheless, long-term systemic antibiotic therapy may lead to the development of resistance and side effects affecting organs. Moreover, antibiotics cannot reach the osteomyelitis site at a sufficient concentration, resulting in limited efficacy and poor patient compliance (Orhan et al., 2006). For the reconstruction of local bone defects, autografts, allografts, masquelet membrane induction technology, and bone transfer technology are currently the most effective methods (Cui et al., 2017; Ye et al., 2018a; Shao et al., 2018). However, they are also associated with problems, such as the need for extra surgery, increased hospitalization time, donor site morbidity, and the occurrence of stress fractures (Keller et al., 2017; Radwan et al., 2020). Therefore, the development of new biomaterials to replace traditional therapeutic methods has become a research hotspot in recent years (Topsakal et al., 2018).

The design and development of a variety of bioactive materials with antimicrobial functions, such as chitosan (CS), silver nanoparticles, magnesium oxide and bioactive glass, have provided a new and promising direction for the treatment of infected bone defects (Galarraga-Vinueza et al., 2017; Shuai et al., 2017). Currently, synthetic and natural materials are commonly used in this setting. These materials offer the advantages of biocompatibility and biodegradability, high porosity, as well as the ability to effectively induce new bone formation (Abueva et al., 2017). Among natural materials, CS (an emerging biological material) has been increasingly used in bone tissue engineering due to its biological and structural similarities with natural tissues (Mostafa et al., 2017).

CS is a biodegradable and biocompatible natural polymer mainly produced by acetylation of chitin, one of the most abundant polysaccharides in nature obtained from the exoskeleton of crustaceans (Kjalarsdóttir et al., 2019; Saravanan et al., 2019). It has a natural polysaccharide structure similar to that of glycosaminoglycan sulfate, which is one of the main components of collagen fibers in the extracellular matrix (ECM). This feature enables CS to provide a microenvironment for cell proliferation and ECM, and has the potential to promote bone formation (Yu et al., 20132013; Zang et al., 2017). Owing to the positive charge on its amino group, CS can bind to the cell membrane, thereby providing the appropriate conditions for cell adhesion (Deepthi et al., 2016; Tang et al., 2020). Moreover, following the depolymerization of CS, chitooligosaccharides with biological activity and improved antibacterial properties are produced. Its monomer product (glucosamine) can be metabolized or excreted from the body (Aam et al., 2010); it also promotes wound healing and hemostasis and reduces inflammation (Shi et al., 2010). In addition, CS can be modified by quaternization, carboxylation, sulfation, and phosphorylation to improve its solubility, antibacterial properties, and chelating ability. It can also be combined with organic materials to improve its biocompatibility and biodegradability, as well as inorganic materials to improve its antibacterial properties. This flexibility renders CS a promising new material for the treatment of infected bone defects (Shin et al., 2005; Venkatesan et al., 2014; Zhou et al., 2017). Given the above properties, numerous studies have investigated the application of CS-based biomaterials for the treatment of infection and promotion of osteogenesis. These studies have demonstrated the ability of CS to induce the repair of infected bone defects (Shi et al., 2010; Covarrubias et al., 2018).

**SCHEME 1:**
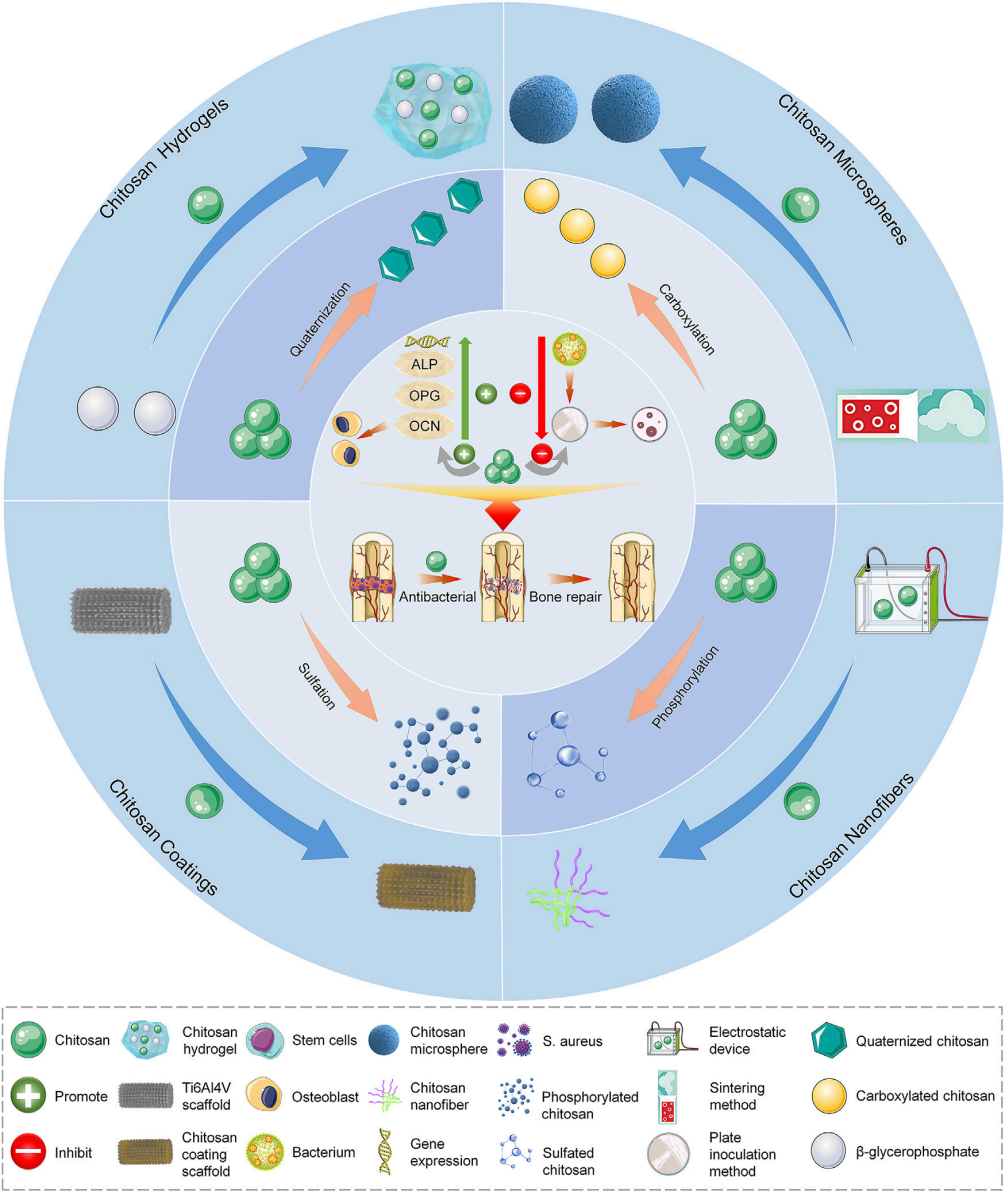
Repair of infected bone defects with different forms and modification methods of chitosan. ALP, alkaline phosphatase; OCN, osteocalcin; OPG, osteoprotegerin; *S. aureus*, *Staphylococcus aureus*.

In this review, we discuss the specific mechanisms underlying the antibacterial properties and osteoconductivity of CS. Also, the advanced strategy for improving these functions of CS, which is essential for the application of CS-based biomaterials, is analyzed. Furthermore, considering the different material forms, we summarize the various existing CS-based biomaterial scaffolds utilized in the treatment of infected bone defects (Scheme 1). Finally, the shortcomings of CS in bone tissue engineering and the prospects of its derivatives and composite materials in medical applications are discussed. This review provides an advanced strategy and theoretical basis for the treatment of infected bone defects with CS-based biomaterials.

## 2 Functionality of CS for Infected Bone Defects

### 2.1 Chemical Structure

CS is derived from chitin and is a linear, semi-crystalline polysaccharide composed of (1→4)-2-acetamido -2-deoxy-b-D-glucan (N-acetyl D-glucosamine) and (1→4)-2-amino-2-deoxy-β-D-glucan (D-glucosamine) units (Deepthi et al., 2016; Islam et al., 2016). CS can be prepared by deacetylation of NaOH and borohydride, or it can be deacetylated by sophisticated grinding of chitin powder. After deacetylation into CS, the viscosity of CS was increased. In addition, the reaction conditions are mild, and the methods are environment-friendly and low cost (Muzzarelli et al., 2014). Through deacetylation, the acetamide group in chitin is converted to the primary amine group to produce CS. The structure of CS is similar to that of cellulose. However, different from cellulose, the hydroxyl group of cellulose C-2 is replaced by the acetamide group in the structure of CS. Due to the effect of the -NH_2_ group at the C2 position, the polymer can be dissolved in acidic solutions; however, the solubility in water is poor. This hydrophobicity is determined by the main polysaccharide chain and the N-acetyl group at the C2 position (Tao et al., 2020a).

The deacetylation degree (DD) is often used to express the number of amino groups in CS; only a DD >60% denotes the production of CS from chitin (Croisier and Jérôme, 2013). CS is the only positively charged polysaccharide in nature, and its charge density depends on the DD and pH values. The DD also has an impact on the biocompatibility of CS. For instance, a higher DD increases the number of positive charges and the interaction between CS and cells, resulting in improved biocompatibility (Kadouche et al., 2016; Aguilar et al., 2019).

### 2.2 Antibacterial Property

As mentioned above, two cationic units constitute the main part of CS. The different proportions of these units affect the molecular weight (MW), DD, and acetylation mode of CS, and determine the strength of its antibacterial properties (Tan et al., 2014). As an antibacterial material, CS has inherent activity and high effectiveness against a variety of bacteria, such as *Escherichia coli* (*E. coli*) and *Staphylococcus aureus* (*S. aureus*), as well as filamentous fungi and yeast (Li and Zhuang, 2020). The antibacterial properties of CS mainly include three aspects. Firstly, the positively charged CS molecule reacts with the negatively charged bacterial molecule on the cell surface. This reaction changes the permeability of the cell, prevents the entry of the substance into the cell or leakage of the substance from the cell, and inhibits its metabolism. This process results in bacterial death (Raafat et al., 2008). Secondly, CS can bind to bacterial DNA and inhibit the synthesis of proteins expressed by bacterial genes. It can also adsorb electronegative substrates in microbial protein cells to disrupt the physiological activities of microorganisms and lead to cell death (Fei Liu et al., 2001). Thirdly, through the metal chelation mechanism, CS inhibits the absorption of basic elements required for the growth of microorganisms and combines the metal ions required by microorganisms to achieve the purpose of antibiosis (Chung et al., 2011).

The antibacterial properties of CS are dose-dependent and influenced by pH. This is because CS can be dissolved only in acidic solutions and becomes polycationic when the pH is < 6.5 (Lim and Hudson, 2004). Ultra-high-MW CS (MW > 10^6^) with different DD (range: 51.04–100%) have been used to test the antibacterial performance against *E. coli* and *S. aureus*. The results showed that CS had stronger antibacterial activity under acidic conditions versus neutral and alkaline conditions. Importantly, it demonstrated its best antibacterial activity when the pH was 6, due to the formation of cation NH^3+^ by the amino groups in CS through protonation under acidic conditions. Under the condition of pH 6.0, the antibacterial activity of CS was gradually increased in parallel with the increase in the DD (Li et al., 2016). Moreover, CS exhibits varied antibacterial activity against different strains. The negative charge on the surface of Gram-negative bacteria is higher than that of Gram-positive bacteria, thereby increasing the adsorption of CS on the surface. Peptidoglycan and phosphoric acid are also present in the cell membrane of Gram-positive bacteria. Therefore, the inhibitory effect of CS on Gram-negative bacteria is stronger than that observed against Gram-positive bacteria (Chung et al., 2004; Rashki et al., 2021). Temperature can also affect the antibacterial property of CS. In the range of 4–37°C, the inhibitory effect of chitosan on *E. coli* will augment with the increase of temperature. This is due to the influence of low temperature on the binding sites of chitosan and cells (Tsai and Su, 1999). Moreover, the temperature also affects the MW of CS, and the antibacterial activity of CS with different MW is also different (Li and Zhuang, 2020). When the MW is below 300 kDa, with the increase of MW, the inhibitory effect of CS on *S. aureus* is enhanced, but the phenomenon of *E. coli* is just the opposite. The inhibition mechanism of CS with high MW and low MW is different. CS with high MW forms a film on the surface of *S. aureus*, which hinders its nutrient absorption, while low MW of CS directly enters the cells of *E. coli* and disturbs cell metabolism (Zhang and Zhu, 2003).

### 2.3 Bone Repair Promotion

The repair of bone defects depends on many factors, such as the proliferation of bone progenitor cells and bone growth factors (Wang et al., 2018a; Munhoz et al., 2018). In bone tissue engineering, bone substitutes play an important role in supporting cell adhesion, growth, and proliferation at the injury site. As mentioned above, CS is similar to the natural ECM component glycosaminoglycan, which creates a local microenvironment for cell growth and supports the proliferation, differentiation, and mineralization of osteoblasts (Pattnaik et al., 2011). Cell adhesion to CS depends on the DD; higher DD values are linked to greater cell adhesion to the surface (Mao et al., 2004). *In vitro* studies have shown that CS can promote the adhesion and proliferation of osteoblasts and mesenchymal stem cells (MSC). Electrical stimulation and electroactivity improve the proliferation and differentiation of electrically signal-sensitive cells, such as osteoblasts (Zhao et al., 2015). Through its osteoconductivity property, CS can effectively respond to this electrical stimulation effect, thus promoting the proliferation of osteoblasts. Co-culture of adipose mesenchymal stem cells (AD-MSC) and human umbilical vein endothelial cells (HUVEC) in a CS scaffold promoted the expression of CD31 in HUVEC and osteogenic differentiation of AD-MSC following electrical stimulation (Zhang et al., 2018). In addition, the osteogenic ability can be further enhanced by combining CS with hydroxyapatite (HA). This approach interferes with the mineralization process and osteogenesis signal pathway in response to electrical stimulation (Oftadeh et al., 2018). Moreover, CS can also enhance the growth of human bone marrow mesenchymal stem cells (BMSC) by promoting the expression of genes related to osteogenesis and calcium-binding proteins, such as type I collagen, integrin-binding saliva protein, osteopontin (OPN), osteonectin (ON), and osteocalcin (OCN) (Mathews et al., 2011). In addition to the influence of MSC, the scaffolds prepared by mixing CS and bioactive glass in different proportions do not have negative influence on the cell activity of stem cells derived from periosteum, but also promote the osteogenic activity. Moreover, due to the existence of bioactive glass, the mechanical properties of the composite scaffold have been improved (Gentile et al., 2012). It also has been found that CS nanofibrous scaffolds increase the proliferation and DNA replication of human osteoblasts and induce the expression of alkaline phosphatase (ALP) mRNA (Ho et al., 2014). In addition, studies have shown that N-acetylglucosamine (the degradation product of CS) can promote osteoblast activity and fibrous callus formation, and significantly shorten the healing time of bone defects. It may also be related to the increase in the expression of bone morphogenetic protein (BMP) induced by N-acetylglucosamine (Nagel et al., 2013). CS molecules contain a large number of amino groups. Hence, protonation can occur under acidic conditions. This process leads to interaction with various negatively charged proteins and glycolipids on the surface of red blood cells, increased blood viscosity, and activation of platelet adhesion (Fan et al., 2015). Aggregation enhances the transport of platelets to the blood vessel wall to achieve physiological hemostasis and promote angiogenesis (Kyzas and Bikiaris, 2015). In addition, by freeze-drying method, platelet-rich plasma (PRP) can be added to the CS gel scaffold, this scaffold can be used as a carrier to deliver PRP, realizing the controlled release of growth factors in it. Moreover, PRP contributes to the formation of hydrogel, promotes cell adhesion, proliferation and differentiation, and further enhances osteogenesis and angiogenesis (Busilacchi et al., 2013). Therefore, CS can promote blood vessel formation, thereby enhancing bone repair (Saran et al., 2014).

Moreover, the CS scaffold has both osteoconductivity and biodegradability at the bone defect site, which are the advantages associated with its application in the treatment of local bone defects (Mathews et al., 2011). Therefore, in bone tissue engineering, CS is typically used as an osteoconductive material in combination with other materials to further promote bone regeneration. These biological materials include metal ions, nano-hydroxyapatite (nano-HA), graphene oxide, bioglasses, and biologically active substances, such as growth factors, BMP2, as well as other combinations (Gentile et al., 2012; Jayash et al., 2017a; López Tenorio et al., 2019; Koski et al., 2020). Mixing CS/gelatin scaffold with graphene oxide promotes osteoblast differentiation, augments protein adsorption, mineralization and degradability, and promotes the healing of tibial bone defects (Saravanan et al., 2017). Bioactive glass is non-toxic and has no inflammatory reaction when implanted *in vivo*, and its degradation products can promote cell proliferation and activate osteoblast gene expression. The addition of HA and nano-bioactive glass to the CS/gelatin significantly improved the adhesion and proliferation of MG-63 osteoblast-like cells (Peter et al., 2010). Therefore, CS by itself or in combination with other materials to further improve bone repair performance also promotes bone healing to a certain extent.

## 3 Modification of CS for Improvement of Properties

Although CS has good biocompatibility, its solubility in most solvents is poor (i.e., in neutral or high pH solutions), which markedly limits its application (Zang et al., 2017). Moreover, CS is rapidly degraded *in vivo* (Tan et al., 2013). The above characteristics compromise the ability of CS to fully exert its effect. Therefore, an increasing number of studies have focused on introducing groups into the side chain of CS or modifying it by copolymerization with polymers to comprehensively improve its activity. The currently used chemical modification methods for CS mainly include quaternization, carboxylation, sulfation, and phosphorylation (Wu et al., 2016). Here, we summarize the advantages of modified CS and its application in the treatment of infected bone defects (Table 1).

**TABLE 1:** Summary of chemical structure, advantages, disadvantages, and main findings of chitosan and its derivatives.

Chitosan and Derivatives	Chemical Structures	Advantages	Disadvantages	Main Findings	References
CS	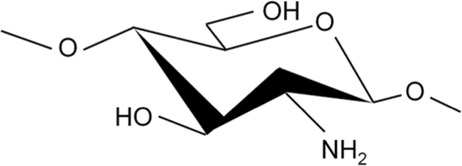	Antibacterial, osteoconductivity, low cost, biocompatibility, biodegradability	Poor water solubility, minimal osteoinductive ability under neutral and alkaline conditions	Promote the proliferation of human osteoblasts by promoting the expression of OPN, OCN and ALP genes	Ho et al. (Ho et al., 2014), Ye et al. (Ye et al., 2018b)
TMC	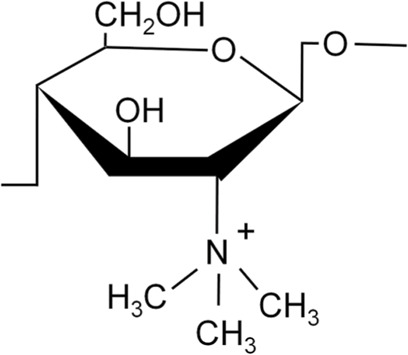	Better water solubility than CS in a wide pH range, enhanced antibacterial and absorption properties	Cytotoxicity at high molecular weight and high degree of substitution	Delivery of osteoprogenitor cells as one of the components of periosteum-mimetic scaffolds	Wu et al. (Wu et al., 2016), Romero et al. (Romero et al., 2015)
HACC	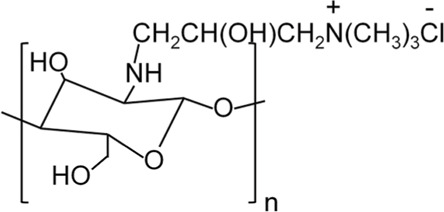	Better antibacterial and water solubility than CS in a wide pH range	Cytotoxicity at medium degree of substitution	Combining with 3D printed scaffolds showed dual functions of antibacterial and osteogenesis both *in vitro* and *in vivo*	Yang et al. (Yang et al., 2016), Yang et al. (Yang et al., 2018)
CMC	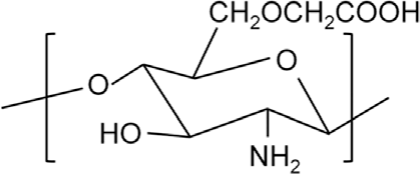	Higher antibacterial activity compared to CS, good water solubility	Mechanical properties may be insufficient	Combining with copper-containing scaffolds to promote bone repair and remove bacteria	Lu et al. (Lu et al., 2018), Shi et al. (Shi et al., 2009)
SCS	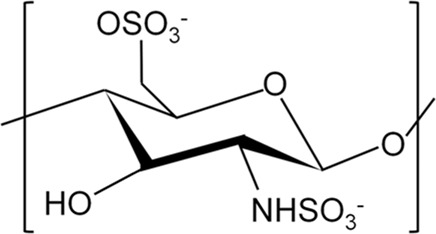	Low cytotoxicity, structure similar to heparin, which enhanced the bioactivity of BMP2	Osteogenic properties may be insufficient when used alone	As a carrier to enhance BMP2 activity, promoting osteoblast proliferation *in vitro* and new bone formation *in vivo*	Zhou et al. (Zhou et al., 2009), Cao et al. (Cao et al., 2014)
PCS	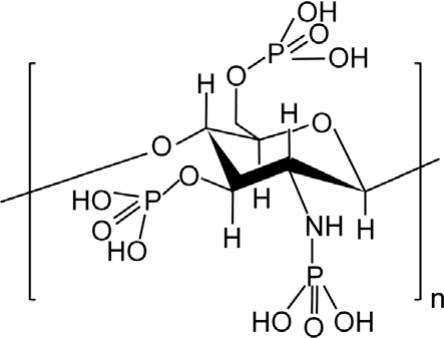	Water solubility with a high degree of substitution, improving mineralization ability by chelating Ca^2+^	Poor antibacterial property	Repair of rabbit ulna defect as a composite scaffold for promoting osteogenesis and inhibiting bone resorption	Tang et al. (Tang et al., 2011), Chen et al. (Chen et al., 2017)

### 3.1 Quaternized CS (QCS)

QCS is prepared by introducing quaternary ammonium groups into the dissociated hydroxyl or amino groups of CS (Ji et al., 2009). Compared with CS, QCS has increased water solubility and a permanent positive charge. There are two common types of QCS, namely N-(2-hydroxyl)propyl-3-trimethylammonium chitosan chloride (HACC) and N,N,N-trimethyl chitosan (TMC) (LogithKumar et al., 2016; Wang et al., 2018b). HACC is composed of CS and glycidyl trimethylammonium chloride dissolved in distilled water at 80°C and subsequently concentrated and lyophilized. TMC is formed by CS and methyl iodine under the catalysis of sodium hydroxide at 60°C (Yang et al., 2015). Other types of QCS include N-(2-hydroxyl) propyl-3-triethylammonium CS chloride and N-(2-hydroxyl-phenyl)-N,N-dimethyl CS (Yang et al., 2015).

#### 3.1.1 HACC

**FIGURE 1:**
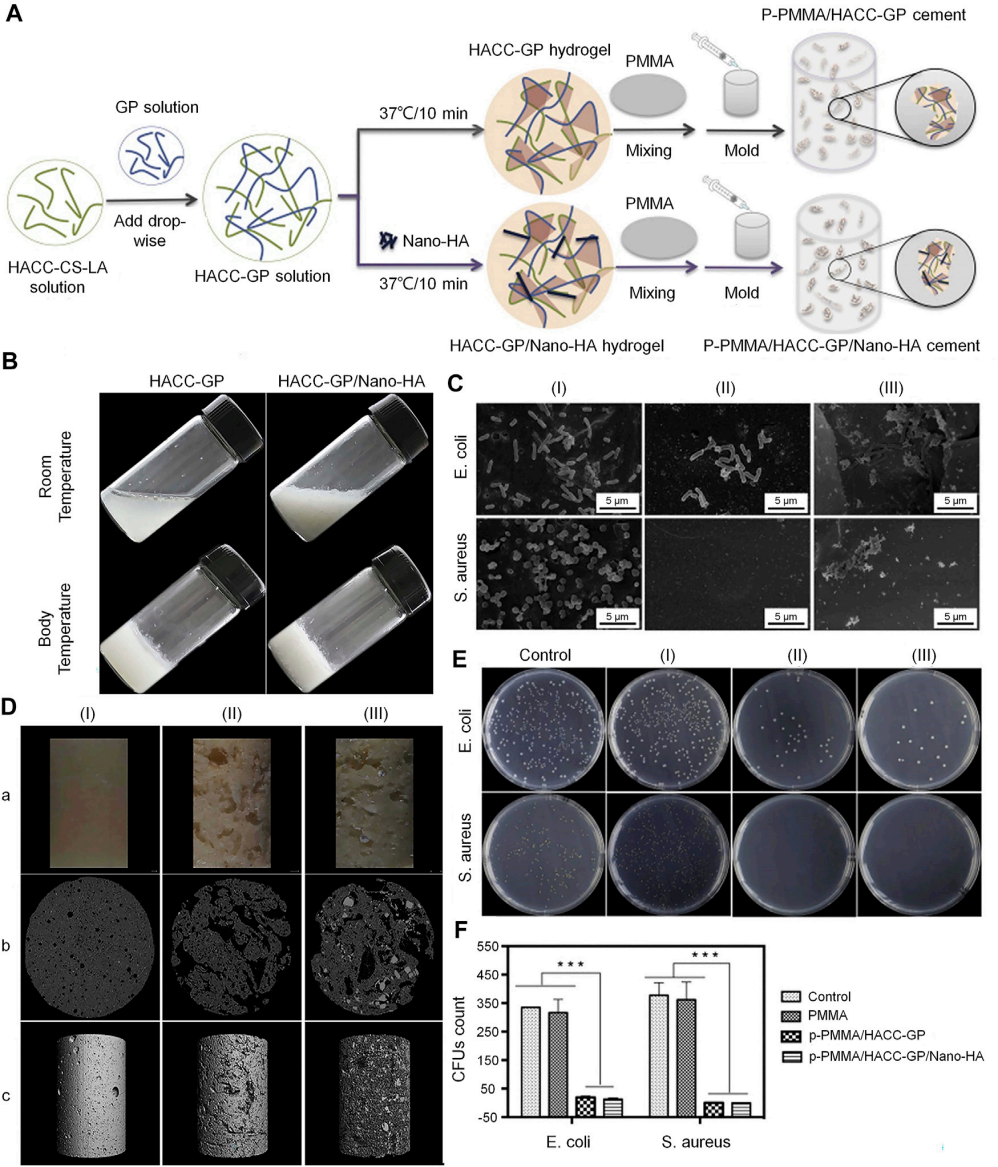
Incorporation of PMMA cement into a HACC-based hydrogel to form a new composite system with dual function for the treatment of infected bone defects (Wang et al., 2018b). **(A)** Synthesis of PMMA-based cement. **(B)** Morphological external phase of HACC-GP and HACC-GP/Nano-HA hydrogel at room temperature and body temperature. **(C)** Bacteriostatic results based on scanning electron microscopy (SEM). **(D)** Surface morphologies (a) and μ-CT observations (b, c) of PMMA-based cements. **(E**,**F)** Agar plate diffusion and colony count results of PMMA-based cements against *E. coli* and *S. aureus.* CFU, colony forming unit; CS, chitosan; *E. coli*, *Escherichia coli*; GP, glycerophosphate; HA, hydroxyapatite; HACC, N-(2-hydroxyl)propyl-3-trimethylammonium chitosan chloride; LA, lactic acid; μ-CT, micro-computed tomography; PMMA, polymethylmethacrylate; *S. aureus*, *Staphylococcus aureus*.

HACC is a commonly used biomaterial for the treatment of infected bone defects. It has exhibited strong antibacterial activity against *E. coli* and *S. aureus* (Tan et al., 2013). Different proportions of CS and glycidyl trimethylammonium chloride determine the degree of substitution of HACC. As mentioned above, an increase in the DD in the CS structure increases positive charge density, allowing it to have a higher electrostatic interaction force (Chou et al., 2015). A higher permanent positive charge density on the main chain of HACC strengthens its binding force with the negative charge on the surface of bacteria (Sajomsang et al., 2009). HACC can be electrostatically combined with the bacterial membrane, resulting in a significant reduction in the risk of bacterial resistance. In addition, the physical state and MW also affect the antibacterial activity of HACC. Low-MW HACC easily penetrates the bacterial cell wall to inhibit mRNA synthesis and DNA transcription (Lu et al., 2016; Crismaru et al., 2011). Also, it has been demonstrated that the water solubility of HACC was increased in parallel with the increase in the degree of substitution. This effect may be due to the quaternary ammonium group with steric hindrance and excellent hydration ability, which greatly reduced the intermolecular and intramolecular hydrogen bonds of CS. The enhancement of water solubility can improve the antibacterial effect. Tan et al. (Tan et al., 2012) successfully prepared the HACC-β-glycerophosphate (β-GP)/nano-HA hydrogel through the addition of polymethylmethacrylate (PMMA) to HACC. This construct demonstrated antibacterial properties against methicillin-resistant *S. aureus*. Furthermore, the existence of the hydrogel endows the system with porosity and mineralization ability. It was also found that HACC-loaded PMMA can prevent the formation of biofilm by staphylococci and downregulate the expression of virulence genes in antibiotic-resistant staphylococci (Figure 1) (Wang et al., 2018b). Grafting HACC onto a three-dimensional (3D) printed scaffold composed of poly (lactic-co-glycolic acid) (PLGA) and HA led to both antimicrobial and osteogenic properties *in vitro* (Yang et al., 2016). In subsequent experiments, researchers established models of infected femoral shaft and femoral condyle defects in rats and rabbits, respectively. HACC-grafted PLGA/HA scaffolds exhibited significantly enhanced anti-infection and bone regeneration capabilities in both models of infected bone defects (Yang et al., 2018). However, the increase in the degree of substitution may have a toxic effect on the cells. Additionally, the toxic effect of HACC concentration on cells should be also considered.

Higher degrees of substitution in HACC increase cytotoxicity. The toxic effect of HACC on cells may be related to mitochondrial damage. HACC may cause acute damage to cells by binding to the mitochondrial membrane at a high concentration. Interaction of HACC with negatively charged cellular components and proteins may also be one of the mechanisms underlying this toxicity (Fischer et al., 2003). Xia et al. (2019) found that low-concentration HACC (0–0.2 mg/ml) stimulated the growth and metabolism of mitochondria. However, when the concentration of HACC was increased to 0.4 mg/ml, it inhibited mitochondrial metabolism; this effect is linked to the decoupling phenomenon.

The optimal drug concentration range of HACC also varies under different degrees of substitution. HACC with a substitution degree of 18% was soluble at pH 12–13. The minimum inhibitory concentrations (MIC) against *Staphylococcus epidermidis* (*S. epidermidis*) and *S. aureus* were 32 and 64 μg/ml, respectively. However, it was not toxic to L-929 cells even at a concentration markedly exceeding its MIC (i.e., 2.5 mg/ml) (Peng et al., 2010; Peng et al., 2011). Another study found that when the degree of substitution of HACC was 40%, the MIC of HACC for *E. coli* and *S. aureus* was 40 μg/ml; the half lethal concentration of AD-MSC was 2.67 mg/ml (Zhao et al., 2015). When the degree of substitution of HACC was increased to 44%, its MIC against *S. epidermidis* and *S. aureus* were 16 and 32 μg/ml, respectively, which was similar to 40% HACC. However, slight toxicity to L-929 cells was noted at the concentration of 2.5 mg/ml (Peng et al., 2010; Peng et al., 2011). At the degree of substitution of 66%, HACC at a concentration of 720 μg/ml can kill half of AD-MSC (Zhao et al., 2015). Although Wang et al. showed that HACC with the degree of substitution ranging 95–98% can play an antibacterial role at a micro concentration of 40, 200, and 1,000 μg/ml against *S. aureus* and *E. coli*, HACC at 200 and 1,000 μg/ml simultaneously showed significant cytotoxicity (Wang et al., 2019).

**FIGURE 2:**
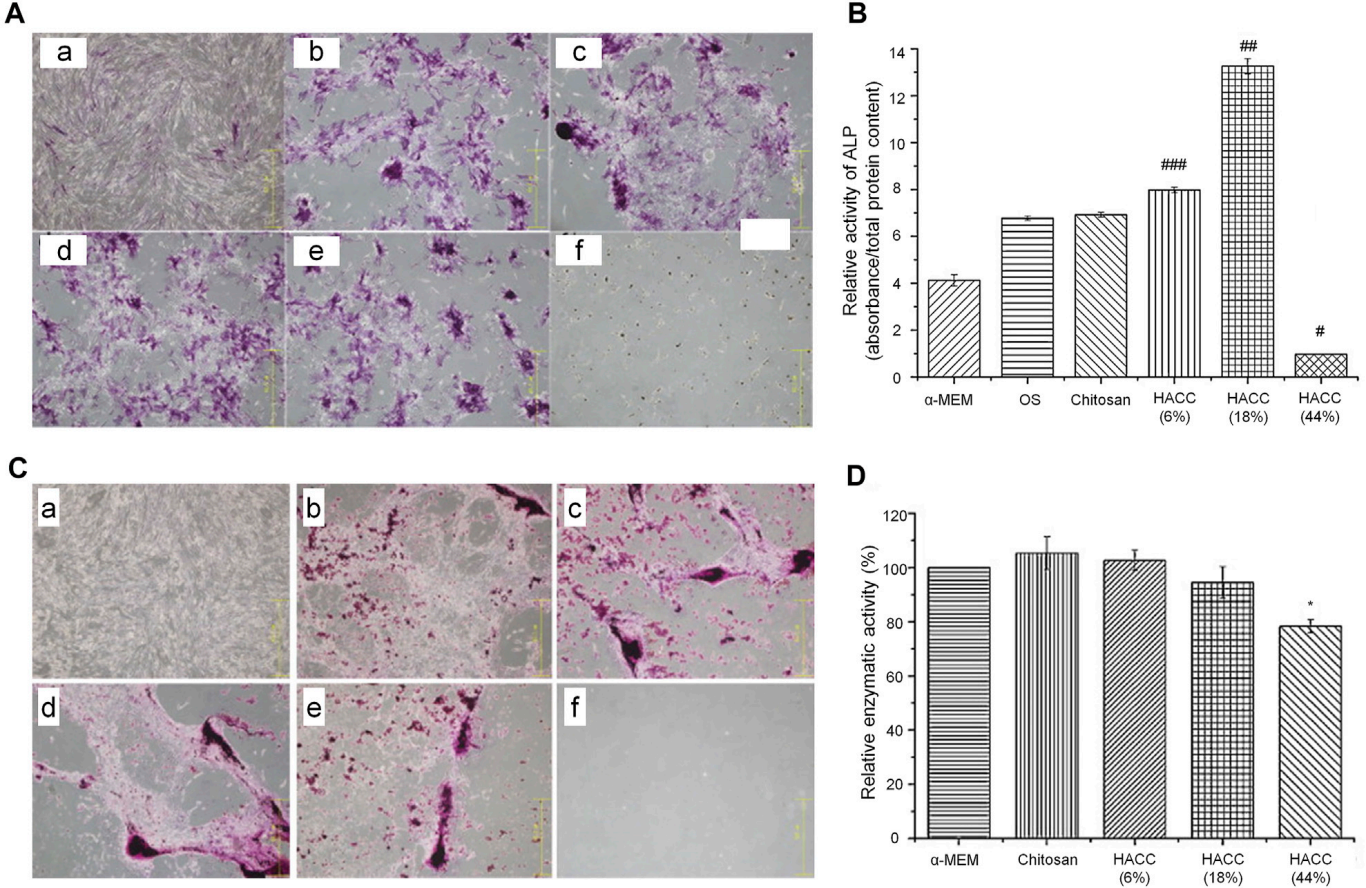
Effects of HACC with different degrees of substitution on cell proliferation and differentiation (Peng et al., 2010). **(A)** ALP staining. (a) α-MEM; (b) osteogenic induction medium (OS) only; (c) chitosan; (d) HACC 6%; (e) HACC 18%; and (f) HACC 44% treated hMSC after 14 days. **(B)** Relative ALP activity in each group after 14 days of culture (^###^ *p* < 0.05, ^##^ *p* < 0.01). **(C)** Alizarin red staining results after 28 days of culture. (a) α-MEM medium only; (b) OS; (c) chitosan; (d) HACC 6%; (e) HACC 18%; and (f) HACC 44%. **(D)** MTT test results for L-929 cells after 48 h of exposure (*p < 0.05). ALP, alkaline phosphatase; α-MEM, α-Minimum Essential Medium; HACC, N-(2-hydroxyl)propyl-3-trimethylammonium chitosan chloride; hMSC, human mesenchymal stem cells; MTT, 3-(4,5-dimethylthiazol-2-yl)-2,5-diphenyltetrazolium bromide.

According to the above evidence, compared with simple low-MW CS, the MIC of HACC was significantly decreased with the increase in the substitution degree from 18 to 98%. However, the minimum bactericidal concentration did not change significantly, indicating that the degree of substitution did not affect it (Shagdarova et al., 2019). When the degree of substitution exceeds 44%, the antibacterial activity may be very strong. This indicates that HACC with a high degree of substitution only requires a low dose to exert its antibacterial effect without inducing toxic effects on cells. However, when the substitution is excessively low (<18%), a very high concentration is needed to produce a satisfactory antibacterial effect. Therefore, the optimal concentration of HACC needs to be analyzed under different degrees of substitution. In an optimal range of the degree of substitution, there is no need to consider the toxic effect of excessive concentrations on cells or the insufficient antibacterial effect of low concentrations. According to the above research results, we conclude that the most suitable range for the degree of substitution in HACC is 18–20%. In this range, HACC has optimal biocompatibility that does not cause toxic effects on cells at the concentration of 2–2.5 mg/ml and also has a sufficient antibacterial effect. The MIC of HACC with a 44% degree of substitution is markedly lower than the concentration at which toxicity to cells was initiated. Nevertheless, the results of alizarin red staining and ALP activity test showed that HACC does not have osteogenic properties and cannot be used as the optimal degree of substitution (Figure 2). Moreover, the critical degree of substitution for HACC is approximately 90%; when the degree of substitution is >90%, toxicity to cells has been initiated before the occurrence of bacteriostasis.

#### 3.1.2 TMC

TMC, one of the most easily synthesized and common forms of QCS, can be synthesized by two methods, namely direct quaternary ammonium substitution, and N-alkylation (Xu et al., 2010). Compared with HACC, TMC has higher antibacterial activity against *E. coli* and *S. aureus*; it has also exhibited some antibacterial effect on *Candida albicans*. The reported MIC against *E. coli* and *S. aureus* were 0.04 and 0.16 mg/ml, respectively, which are markedly lower than that of HACC. The antibacterial activity of TMC increases in parallel with an increase in the length of the alkyl substituent chain. When TMC was further quaternized to N,N,N-trimethyl O-(2-hydroxy-3-trimethylammonium propyl) CS, it showed higher antibacterial activity than TMC (Jia et al., 2001; Huang et al., 2013). A study showed that novel electrospun silver nanoparticles loaded with nanofiber mats containing TMC, polyacrylic acid, or poly (2-acrylamide-2-methylpropanesulfonic acid) have better antibacterial activity against *E. coli* and *S. aureus* than the film without TMC and silver nanoparticles (Kalinov et al., 2015). The antibacterial and polycation properties of TMC can be used to develop polyelectrolyte complexes and improve the modification strategy; hence, more application prospects should be explored. Romero et al. showed that a 71% degree of substitution of TMC can be used with heparin as a periosteal mimic to form a multilayer membrane coating to cover the cortical bone for the promotion of bone healing; in this coating, heparin and TMC acted as polyanion and apolipoprotein, respectively. TMC did not have a toxic effect on AD-MSC and exerted a strong antibacterial effect. This experiment confirmed the potential usefulness of TMC for the development of polyelectrolyte complexes (Romero et al., 2015). While the antibacterial capabilities of TMC have been extensively studied, further work is needed to investigate its polycationic nature. Such research would expand its applicability as a growth factor delivery vehicle in the treatment of infected bone defects.

### 3.2 Carboxylated CS

**FIGURE 3:**
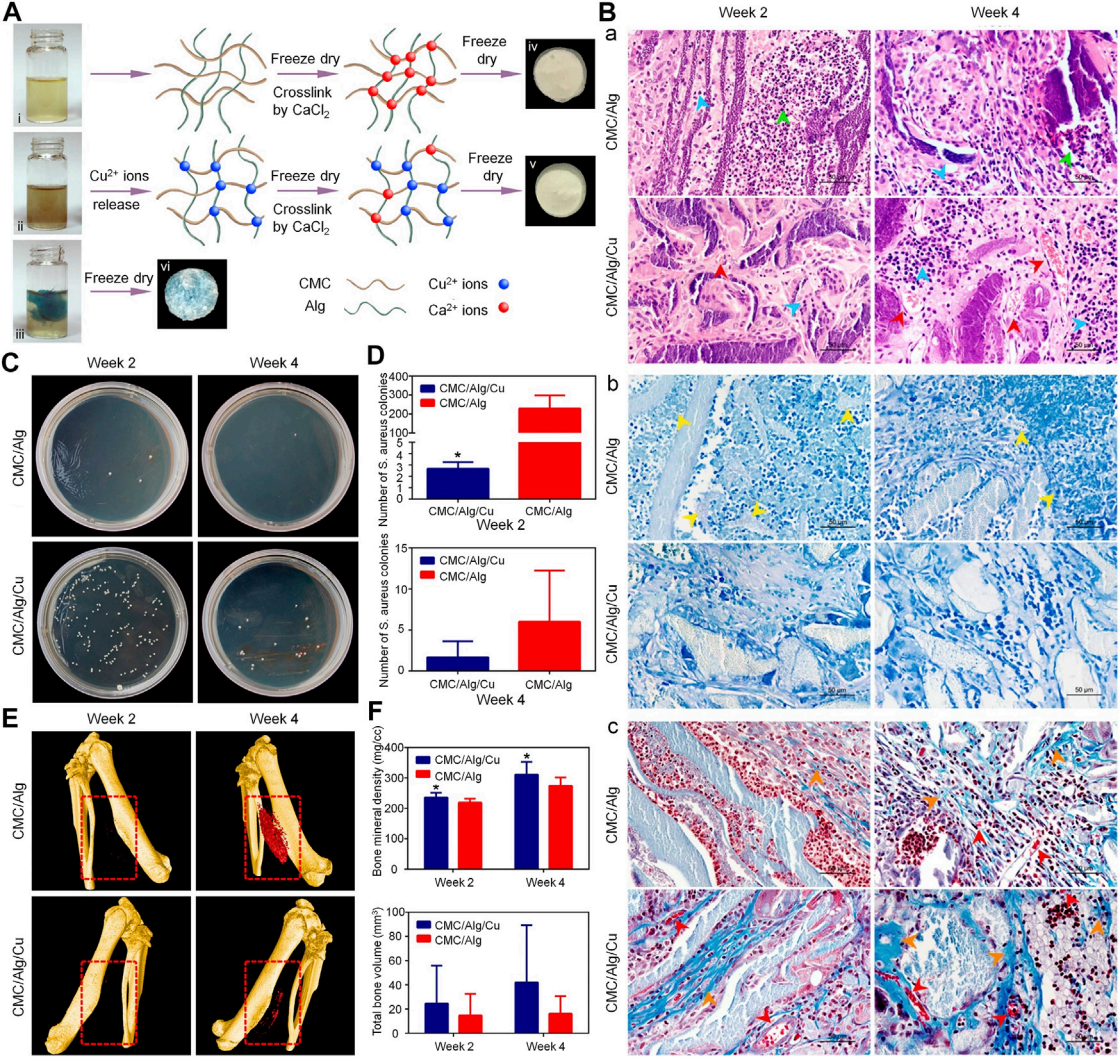
Treatment of infected bone defect with a copper-containing CMC/sodium alginate scaffold (Lu et al., 2018). **(A)** Schematic of the scaffold preparation. i, ii, and iii denote CMC/Alg solution, CMC/Alg solution with Cu nanoparticles, and Cu^2+^ solution with CMC/Alg, respectively. **(B)** H&E (a), Giemsa (b), and Masson’s trichrome (c) staining results at 2 and 4 weeks after implantation. **(C,D)** The antibacterial properties of the scaffolds *in vivo*. *p < 0.05. **(E)** The μ-CT results at 2 and 4 weeks after implantation. **(F)** Quantitative analysis of bone regeneration. Alg, alginate; CMC, carboxymethyl chitosan; μ-CT, micro-computed tomography; H&E, hematoxylin and eosin.

Most studies on carboxylated CS have focused on carboxymethylation reactions. CS and monohalocarboxylic acid can produce different types of carboxymethyl chitosan (CMC) under different reaction conditions. Common carboxymethyl derivatives are O-CMC, N,O-CMC, N-CMC, and N-succinyl CS (Shi et al., 2006). Similar to QCS, the water solubility of CMC in various pH environments is also controlled by the degree of carboxymethylation (Chen et al., 2004). CMC also has antibacterial properties. However, the degree of substitution of CMC has a minimal impact on its antibacterial activity, which mainly depends on the number of NH^3+^ groups in the structure (Sun et al., 2006). Although carboxylated CS obtained enhanced antibacterial properties, the effects of different types of CMC on Gram-positive or Gram-negative bacteria differed. For example, O-CMC and N,O-CMC exhibited strong antibacterial activity against *E. coli* and *S. aureus*, respectively (Fei Liu et al., 2001). With the gradual increase in the concentration gradient (i.e., 5, 8, and 10 mg/ml), the antibacterial activity of O-CMC and N,O-CMC was also gradually increased in a dose-dependent manner. Moreover, at the concentration of 0.4, 0.8, and 1 mg/ml, O-CMC and N,O-CMC did not show cytotoxicity to the cells (Anitha et al., 2009). Owing to the existence of the -NH_2_-CH_2_-COOH functional group, CMC has good metal chelating ability. Wahid et al. (2017). I t has been demonstrated that CMC could be used to prepare supramolecular hydrogels with metal ions, such as Ag^+^, Cu^2+^, and Zn^2+^. The gelation process of the hydrogel occurs rapidly and can result in good mechanical properties, particularly after combination with metal ions. Moreover, excellent antibacterial properties against *S. aureus* and *E. coli* were also revealed compared with those of pure CMC. Direct crosslinking of CMC with metal ions may lead to uncontrolled release. This problem can be solved through the addition of other polymers to form a mixture. Adding Cu nanoparticles to the CMC and alginate polymer mixture can enable the controllable release of Cu ions to form scaffolds. *In vivo* and *in vitro* experiments showed that the combination of Cu nanoparticles with a CMC/alginate scaffold could produce excellent antibacterial and osteogenic properties, and did not have a toxic effect on MC3t3-E1 cells (Figure 3) (Lu et al., 2018). Apart from chelating Cu^2+^ and Zn^2+^ to augment the antibacterial properties, CMC can chelate Ca^2+^ to induce apatite deposition and improve osteogenic activity. This effect may be related to the fact that carboxymethyl groups provide more nucleation sites. N,O-CMC and polyphosphate are crosslinked by a Ca^2+^ bridge; this process yielded a scaffold which has exhibited a strong regeneration-inducing activity in rat skull defects (Müller et al., 2015). BMP and other proteins promote the proliferation of bacteria to some extent; nevertheless, the existence of CMC inhibits or even reverses this effect. CMC can also help overcome the problem of biofilm formation by bacteria on the implant surface. Functionalizing the surface of the titanium alloy material with CMC and BMP2 by covalent grafting can significantly inhibit *S. aureus* and *S. epidermidis* adhesion. In addition, CMC does not affect the differentiation and proliferation of osteoblasts (Shi et al., 2009). CMC can also be synthesized with other biomaterials to improve its osteogenic performance in bone tissue engineering. The combination of CMC and gelatin with laponite at the concentration of 10% (weight/weight) can significantly enhance the osteogenic differentiation ability of rat BMSC and has a strong compatibility *in vitro*. Notably, it can promote the healing of rat skull defects *in vivo* (Li et al., 2017).

### 3.3 Sulfated CS (SCS)

CS can be sulfated using sulfuric acid or sulfonic acid salts (Zhang et al., 2015). Unlike the aforementioned two modified CS, SCS is designed to cooperate with BMP2 for the promotion of healing in bone defects. The sulfate group in SCS is similar to natural anticoagulant heparin, which contains BMP2-binding sites. Compared with natural heparin, SCS has stronger activity on BMP2. However, the antibacterial properties of SCS have rarely been reported (Green et al., 2009). Adding SCS to the BMP2-loaded calcium-deficient HA scaffold can increase the cumulative release of BMP2 compared with the scaffold without SCS. This composite scaffold promoted the repair of skull defect in a rat model (Zhao et al., 2012). In another study, 2-N-SCS, 6-O-SCS, and 2-N, 6-O-sulfated chitosan (26SCS) were successfully synthesized. Among them, 26SCS is the most suitable enhancer for BMP2 with a sulfur content of 12.89 ± 14.86%. Low-dose 26SCS (0.625–2.5 μg/ml) stimulates the differentiation of osteoblasts induced by BMP2 *in vitro* and stimulates ectopic bone formation *in vivo*. These effects may be related to the fact that 26SCS can increase the expression levels of noggin (NOG) mRNA. Nevertheless, SCS alone does not promote the osteogenic differentiation of C2C12 myoblast cells. This finding indicated that SCS should work together with BMP2 to indirectly affect osteogenic differentiation (Zhou et al., 2009). The preparation of a photopolymerisable hydrogel incorporating recombinant human BMP2 (rhBMP2)-loaded 26SCS-based nanoparticles can promote the attachment and osteogenic differentiation of human MSC *in vitro*. New bone formation was found in a rabbit model of the radial defect (Cao et al., 2014). Therefore, we conclude that SCS alone cannot be used as a bone-forming material. To achieve this purpose, it needs to be combined with osteogenic biomaterials, such as BMP2. Additionally, the combination of SCS with metal ions or antibacterial substances can be explored to improve the antibacterial properties and serve as a synergistic factor in the regeneration of local infected bone defects.

### 3.4 Phosphorylated CS (PCS)

Compared with CS, ionic conductivity, and swelling index are both improved in PCS. Although the crystallinity was reduced, the tensile strength remained similar to that of CS. Unlike CS, PCS has a significantly rough surface (Jayakumar et al., 2008). PCS is mainly produced through two approaches. The first approach involves the reaction between the CS hydroxyl group and phosphorus pentoxide group in the presence of methanesulfonic acid, which can be used as a protective agent for the CS amino group (Jayakumar et al., 2007). The second approach involves the reaction of CS hydroxyl functional groups with phosphoric acid in the presence of urea (Jayakumar et al., 2008). Owing to the presence of the phosphate group, PCS has metal chelating ability. It can be combined with calcium phosphate crystal particles, thus improving the mechanical properties of PCS and promoting bone properties. By combining PCS and HA at a weight ratio of 30/40, the maximum mechanical property of approximately 70.25 MPa can be obtained; also, most of its original compressive strength can be maintained for 20 days. (Li et al., 2011). Studies on the osteogenic effect of PCS have shown that it modulates the expression levels of osteoclastogenic factors, nuclear factor κB ligand-receptor activator (RANKL), and osteoprotegerin (OPG) protein. Moreover, it may inhibit osteoclast differentiation by upregulating the expression ratio of OPG and RANKL in human primary osteoblasts (Tang et al., 2011). By mixing PCS and disodium (1→4)-2-deoxy-2-sulphoamino-β-_D_-glucopyranuronan (SCS) into PLGA/tricalcium phosphate scaffolds, we can obtain scaffolds with dual functions, namely osteogenesis and inhibition of bone resorption. These scaffolds promoted bone healing in an ulna defect model *in vivo* (Chen et al., 2017). Collectively, these properties ensure that PCS can be used as a potential biomaterial in the treatment of infected bone defects.

### 3.5 Others

This part describes the rarely modified CS; many functions have not been confirmed thus far, and the osteogenesis and antibacterial properties require further investigation. Thiolated chitosan (TCS) can be obtained by adding the thiol group to the primary amino group of CS, which can improve some properties of CS (e.g., good solubility at neutral pH and formation of disulfide bonds with other thiol groups in proteins) (Wang et al., 2020). Compared with the traditional CS/β-GP hydrogel, the TCS/HA/β-GP hydrogel has a higher storage modulus (G’) and loss modulus (G”), as well as a more appropriate degradation rate and low cytotoxicity (Liu et al., 2014).

Succinylation of CS can effectively augment water solubility and biocompatibility and yield new functional groups. The succinylated CS hydrogel can controllably release drugs under the influence of pH. Through combination with bone graft material, this hydrogel can increase the rates of cell growth and bone differentiation. Its mechanical properties, such as compressive strength and Young’s modulus, decrease with the increase in the rate of succinylation (Lee et al., 2020; Lee et al., 2021). Hydroxypropyl CS and hydroxybutyl CS are two forms of hydroxyalkyl CS. Hydroxyalkyl CS was obtained by the substitution reaction of CS and epoxide on amino or hydroxyl groups; however, it is rarely used in the treatment of bone defects (LogithKumar et al., 2016). By grafting maleic acid, hydroxypropyl CS can effectively inhibit >90% of *E. coli* and *S. aureus* within only 30 min. In addition, hydroxybutyl CS can rapidly form a gel, stably exist *in vivo*, and is injectable (Peng et al., 2005; Dang et al., 2006). The ethylene glycol CS has better mechanical properties and a slower degradation rate than pure CS (Huang et al., 2016). The above modified CS is rarely reported. Although its performance has been improved, its synthesis may be more complicated. Further investigation is warranted to improve its performance and render it suitable for the treatment of infected bone defects.

## 4 Application in the Treatment of Infected Bone Defects

In addition to the chemical modification of CS, mixing CS with other materials (e.g., antibacterial and osteogenesis-promoting substances) is an excellent strategy for improving its properties. CS can be used as a carrier for the delivery of antibacterial drugs and osteogenic molecules (e.g., vancomycin, parathyroid hormone, and BMP2), thereby increasing its effectiveness in the treatment of infected bone defects (Wang et al., 2018a; Nancy and Rajendran, 2018). This part summarizes the application of CS-based biomaterials in the treatment of infected bone defects.

### 4.1 Injectable Hydrogels

**FIGURE 4:**
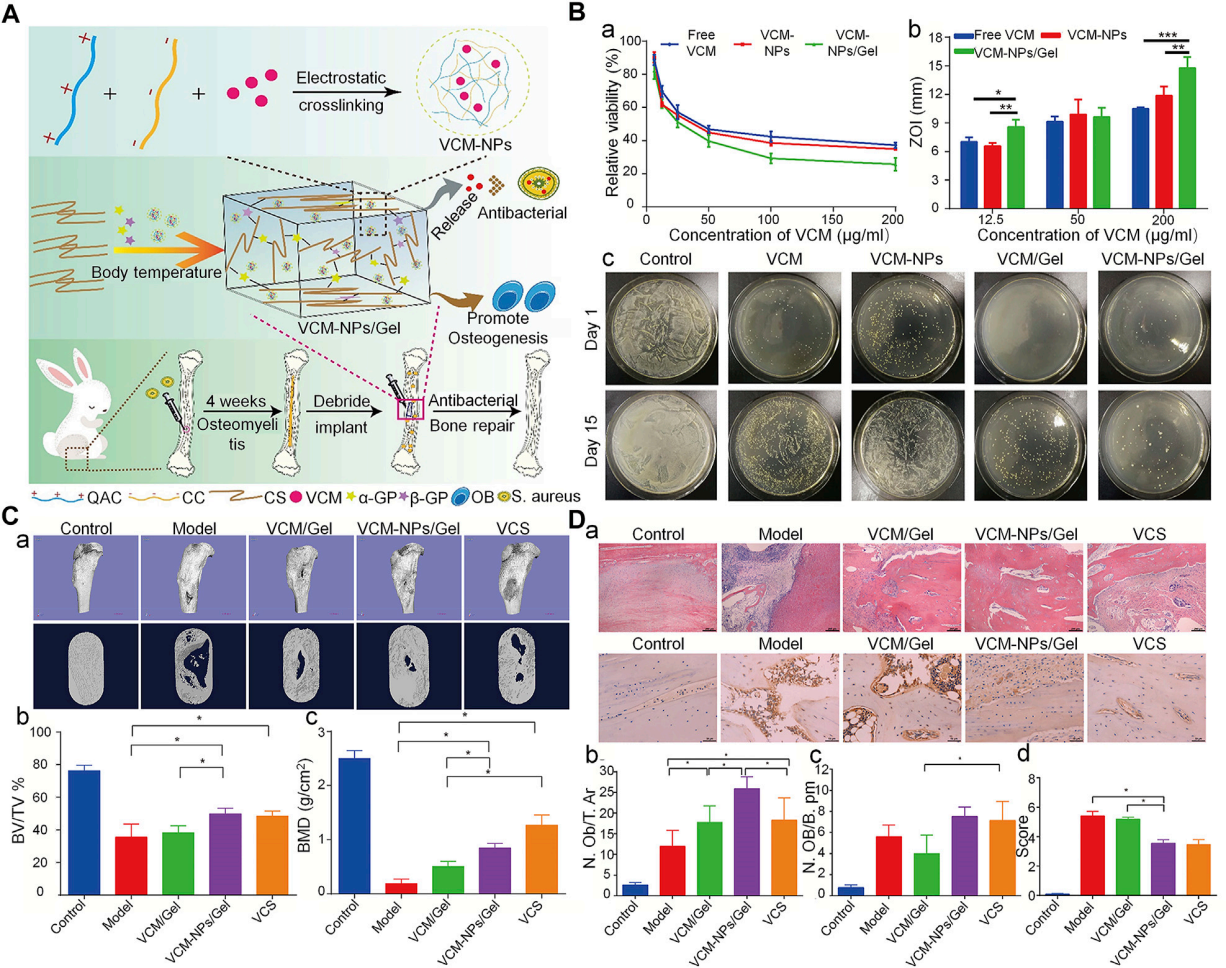
Injectable thermosensitive hydrogel system for the delivery of VCM in the treatment of osteomyelitis (Tao et al., 2020b). **(A)** Schematic diagram of the synthesis of antibacterial and osteogenic hydrogels. **(B)** (a), (b) VCM, VCM-NPs, and VCM-NPs/Gel against *S. aureus* with different concentrations of VCM; (c) Agar plate diffusion of different groups at 1 and 15 days. **(C)** (a) The results of μ-CT bone morphology after treatment with VCM/Gel, VCM-NPs/Gel, and vancomycin-calcium sulfate (VCS); (b), (c) Quantitative analysis of μ-CT in each group. **(D)** (a) Histopathology and IL6 immunohistochemical analysis of the rabbit tibia; (b), (c) Quantitative analysis of new bone formation and (d) Quantitative analysis of IL-6 immunohistochemical staining. (*p < 0.05; **p < 0.01, and ***p < 0.001). BMD, bone mineral density; BV/TV, bone volume/total volume; IL6, interleukin 6; μ-CT, micro-computed tomography; N. Ob/T. Ar, osteoblast number/trabecular bone area; N. OB/B. pm, osteoblast number/bone perimeter; NPs, nanoparticles; *S. aureus*, *Staphylococcus aureus*; VCM, vancomycin; ZOI, zone of inhibition.

The CS hydrogel scaffold has a 3D porous structure, which can simulate the microenvironment of the ECM, promote cell adhesion and proliferation, and allow nutrient and metabolite exchange and cell migration. Moreover, it can encapsulate osteoblasts or growth factors to promote the regeneration of bone tissue (Sultankulov et al., 2019). The CS-based thermosensitive hydrogel has recently attracted attention in bone tissue engineering. CS itself is not a thermosensitive polymer, however, through modification, thermosensitivity can be realized. For example its thermal gelation can achieved by adding GP to the CS solution (Ji et al., 2009). GP is a natural organic compound that exists in the body and one of the components of the osteogenic medium. It can promote the differentiation of MSC into osteoblasts by extracellular related kinases (Wang et al., 2018b). This type of hydrogel can exist in a fluid state at room temperature. Following injection into the body, it forms an *in-situ* stable hydrogel under body temperature conditions (Zhou et al., 2015). This property of the CS hydrogel allows it to be injected directly into the bone defect cavity, regardless of the shape and size. Moreover, changing from the original liquid state to the gel state can make it act as a favorable drug carrier. Therefore, the CS/β-GP injectable hydrogel is considered an outstanding biomaterial for bone reconstruction. Nevertheless, it is also characterized by certain shortcomings, such as mechanical stability, and insufficient osteoconductivity. Some modification methods have been developed to solve this problem. For instance, the CS/GP hydrogel can be used as a carrier of cells, growth factors, and drugs. Using the CS/GP hydrogel, Huang et al. (Huang et al., 2011) added collagen and nano-HA to prepare a stable gel for the delivery of rat BMSC. The gel showed good stability, which may be related to the hydrogen bond formed between collagen and CS. This hydrogel system showed enhanced mechanical properties and ability to induce osteogenesis. To further improve the osteogenic properties of the CS hydrogel, Jayash et al. produced an OPG-CS gel for the treatment of critical-sized skull defects in rabbits. Rabbits in the OPG-CS gel group exhibited more obvious new bone formation at 6 and 12 weeks after treatment compared with those in the CS gel group (Jayash et al., 2017b). Moreover, using the CS/GP hydrogel as a carrier for the delivery of antibiotics can realize an appropriate antibacterial effect. A CS/GP hydrogel containing nanoparticles loaded with vancomycin released the drug continuously for >26 days, inhibiting *S. aureus* both *in vitro* and *in vivo* (Figure 4) (Tao et al., 2020b).

In addition, CS can be modified and mixed with other materials to improve its performance. Single-component polymer hydrogels may have poor mechanical properties and insufficient cell adhesion. Sun et al. (Sun et al., 2021) used polyethylene glycol diacrylate/TCS to form a double-network hydrogel. This dual-network hydrogel has excellent mechanical properties, high crosslinking density, and low swelling degree. In an *in vivo* rat model of skull bone defects, it was demonstrated that addition of BMP2 to this hydrogel can significantly augment new bone formation and biocompatibility. In addition, modification of this hydrogel with thiolated halloysites improves adhesion to MC3T3-E1 cells and further promotes proliferation. This is related to the participation of thiol on the outer surface in the disulfide exchange during the process of cell adhesion, which is mediated by fibronectin and collagen (Rosenberg et al., 2019).

Traditional methods (e.g., crosslinking agents and chemical reactions) for the synthesis of gels may have adverse effects on living cells and biologically active factors. Guanidinylated CS supramolecular hydrogels, which are driven by reversible non-covalent bonds, include ionic interaction and hydrophobic interaction hydrogen bonds, and have been associated with self-healing and injectable properties (Zhang et al., 2020). Laponite acts as a physical crosslinker with osteoinductive properties; the hydrogel enhances cell adhesion and promotes osteogenic differentiation of MSC by activating the Wnt/β-catenin signaling pathway.

### 4.2 Coatings

A major goal in the field of orthopedic implant materials is to provide a bio-interactive surface that can prevent bacterial adhesion and enhance biological activity (Leedy et al., 2011). The osteoconductivity, biodegradability, antibacterial, drug delivery properties, and flexibility of processing and modification of CS render it a potential coating material for orthopedic implants. The conventional preparation processes for CS coating include electrophoretic deposition (EPD), spin coating, electrostatic spinning, and sol-gel methods (Kumari et al., 2021). EPD has become one of the most commonly used methods for the preparation of coatings due to its ability to control the coating composition and complex shape, high efficiency, simplicity, and the absence of a need for a crosslinking agent (Obregón et al., 2019). CS is also highly positively charged; thus, it is easy to prepare a coating using the cathodic EPD method. The use of CS as a coating material has two main functions. Firstly, it can induce calcium deposition on the surface of the coating, thus improving the mineralization ability and promoting osteogenesis. Li et al. (2021) coated the CS/gelatin hydrogel on the poly (aryl ether nitrile ketone)-containing phthalazinone moiety substrate (PPENK) through the spin coating method. Due to the presence of CS, this composite coating can chelate Ca^2+^ to promote the deposition of calcium phosphate at the mineral phase on the surface of the PPENK matrix and enhance the biomineralization potential of the coating, which has exhibited biocompatibility and osteogenic properties for MCET3-E1.

**FIGURE 5:**
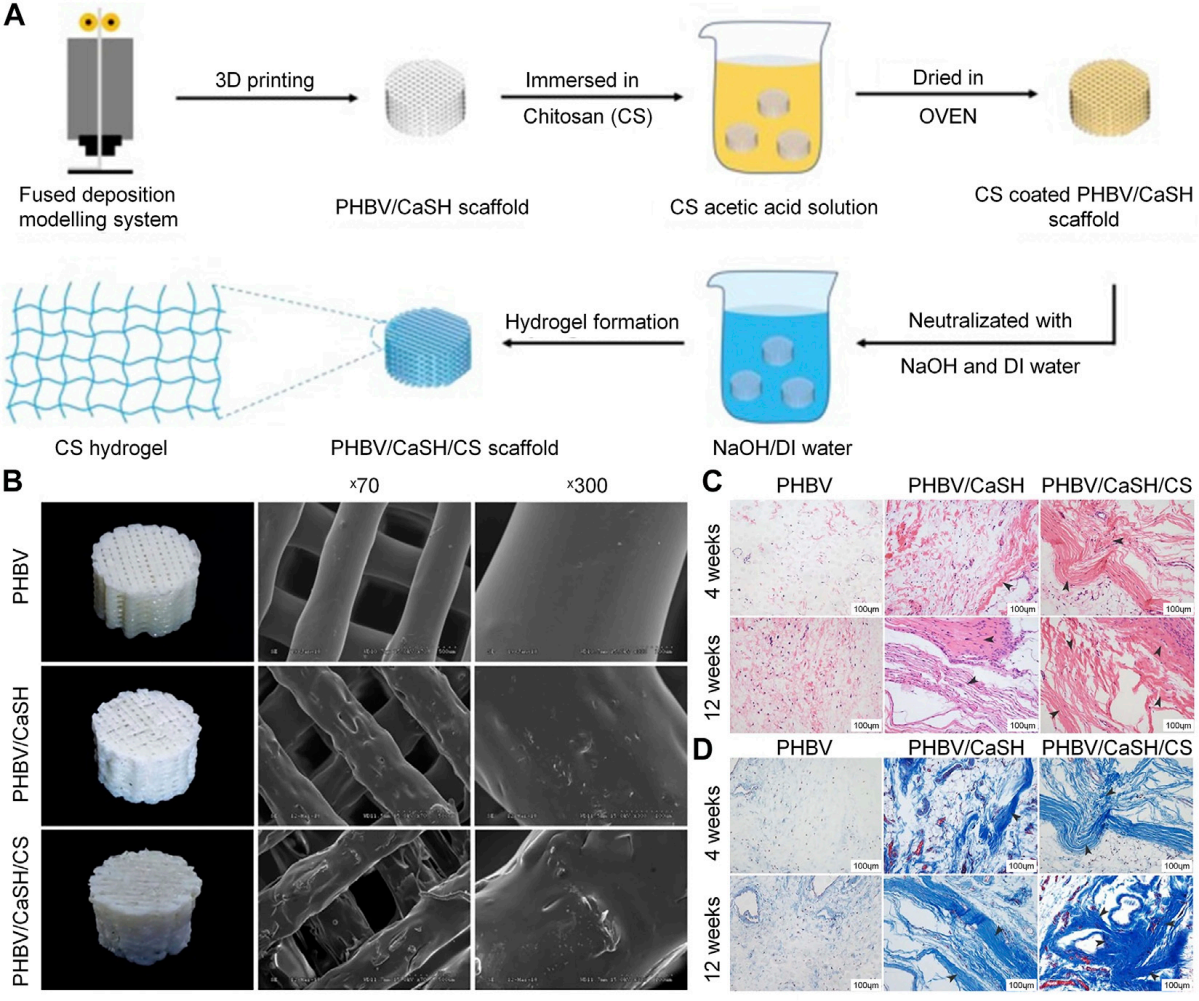
Chitosan-coated PHBV/CaSH scaffold was used to improve the bone repair (Ye et al., 2018b). **(A)** Preparation process for the PHBV/CaSH/CS scaffold. **(B)** Surface morphology and mechanical properties of the PHBV, PHBV/CaSH and PHBV/CaSH/CS scaffolds. **(C)** H&E staining results at 4 and 12 weeks. **(D)** Masson’s trichrome staining performed at 4 and 12 weeks. 3D, three-dimensional; CS, chitosan; DI, deionized; H&E, hematoxylin and eosin; PHBV/CaSH, poly (3-hydroxybutyrate-co-3-hydroxyvalerate)/calcium sulfate hemihydrate.

Secondly, as a carrier for the delivery of antibiotics, biomaterials, and growth factors, the CS coating has many advantages, such as a controllable drug release rate and a slow degradation rate (Frank et al., 2020). Coating CS on titanium alloy scaffold to delivery ciprofloxacin can strongly inhibit *S. aureus*, and does not affect the osteogenic activity of MG63 osteoblast-like cells *in vitro*. In addition, about 77% of total ciprofloxacin was released in 7 days (Mattioli-Belmonte et al., 2014). Beenken et al. (Beenken et al., 2014) demonstrated that CS-coated calcium sulfate containing daptomycin can effectively delay the release of daptomycin and maintain the activity of the released drug for 10 days *in vitro*. In the absence of CS coating, the concentration of daptomycin eluted from the calcium phosphate particles was rapidly decreased. In a rabbit model, calcium sulfate pellets with CS coating exerted a better effect on osteomyelitis than those without CS coating. Nancy et al. (Nancy and Rajendran, 2018) produced a double-layer coating through the EPD method, using the TiO_2_-strontium-incorporated HA as the first layer and vancomycin-added CS/gelatin as the second layer. This double-layer coating has both antibacterial and osteogenic properties, and can sustain the release of vancomycin over 48 h. In bone tissue engineering, porous bioglass is also commonly used as a carrier of osteogenic or antibacterial drugs. However, it is often characterized by burst release of drugs, which shortens the duration of its effectiveness (Soundrapandian et al., 2010). This shortcoming can be overcome by combining CS with bioactive glass, which can effectively prolong the release cycle to prevent the occurrence of osteomyelitis. Patel et al. (2012) synthesized a composite coating with CS and bioactive glass nanoparticles via the EPD method. Ampicillin was eluted continuously from the CS-bioactive glass nanoparticles coating for 10–11 weeks, confirming its ability for long-term drug delivery. Besides CS molecules, calcium and silicon ion products eluted from bioactive glass nanoparticles can promote the osteogenic ability of MC3T3-E1 cells. Furthermore, bioactive glass nanoparticles and CS can jointly promote the formation of apatite on the coating surface and enhance the mineralization ability. Because of their hydrophobicity, most synthetic polymeric materials do not support cell adhesion, proliferation and differentiation. The surface coating formed with CS can enhance the surface hydrophilicity of the polymer material, thereby increasing the cell adhesion (Tong et al., 2011). Coating CS on a 3D-printed poly (3-hydroxybutyrate-co-3-hydroxyvalerate)/calcium sulfate hemihydrate scaffold through a fused deposition modeling approach can improve the osteogenic performance of the scaffold both *in vivo* and *in vitro* (Figure 5). Due to the presence of CS, the scaffold can promote the adhesion and proliferation of rat BMSC, and upregulate the expression of osteogenesis-related genes (e.g., OCN, OPN, and BMP2) to enhance the osteogenic ability (Ye et al., 2018b).

### 4.3 Microspheres

Microspheres are flowing particles typically composed of inorganic or polymer materials. Generally, microspheres are thought to be excellent cell or drug transport vehicles. Cell or drug/microsphere complexes may be implanted directly into the body, which can simplify procedures and improve cell survival (Grellier et al., 2009). Notably, microspheres have a large specific surface area; therefore, modification of their surfaces may promote cell–or drug–substrate contact (Park et al., 2010).

**FIGURE 6:**
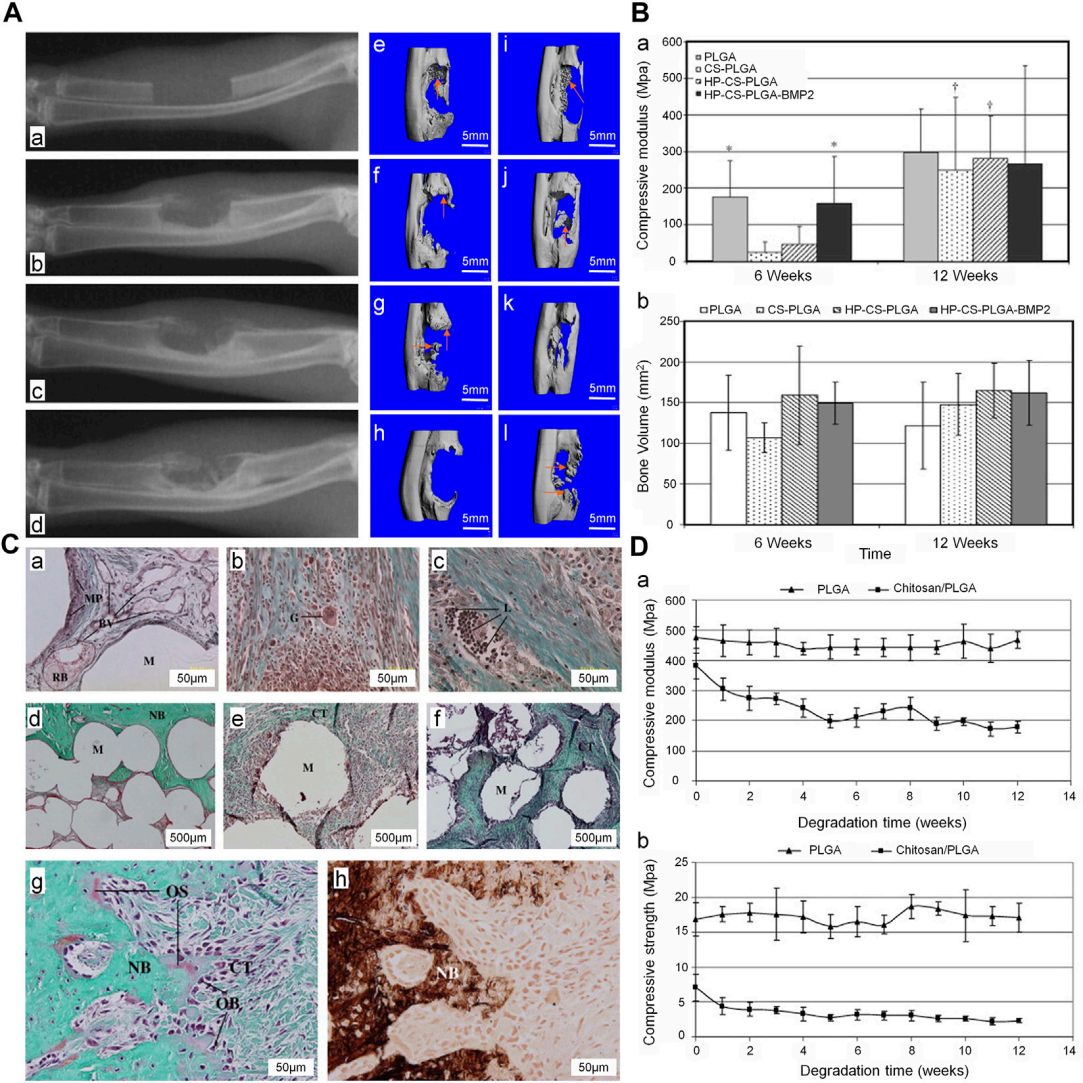
Treatment of ulna defect with CS/PLGA sintered microspheres (Jiang et al., 2010). **(A)** The X-ray images of the ulna defect (a), X-ray (b, c, d) at 4, 8, and 12 weeks after implantation in the HP-CS-PLGA-BMP2 group. Three-dimensional CT, (e–h) and (i–l) at 6 and 12 weeks after implantation in the PLGA, CS-PLGA, HP-CS-PLGA, and HP-CS-PLGA-BMP2 groups, respectively. **(B)** (a) Compressive modulus (b) and bone volume at 6 and 12 weeks after implantation (*p < 0.1). **(C)** Masson’s trichrome staining performed (a) at 6 weeks after implantation in the PLGA group and (b, c) at 6 and 12 weeks after implantation in the HP-CS-PLGA group. Masson’s trichrome staining (d–f) performed at 12 weeks after implantation in the PLGA, HP-CS-PLGA, and HP-CS-PLGA-BMP2 groups. Masson’s trichrome (g) and von Kossa staining of HP-CS-PLGA performed at 12 weeks after implantation at 50 μm. **(D)** Compressive modulus (a) and compressive strength (b) analysis (*p* < 0.05). BMP2, bone morphogenetic protein 2; CS, chitosan; CT, computed tomography; HP, heparin; PLGA, and poly (lactic-co-glycolic acid).

CS has good biocompatibility and biodegradability. Sintering, coagulation precipitation, and emulsion crosslinking methods are the most widely utilized processes for producing microspheres from CS (Huang et al., 2014). Using the sintering microsphere technique, Jiang et al. (2010) produced a CS and PLGA sintered microsphere scaffold. The mechanical properties of the scaffold were within the range of the human trabecular bone, and the degradation rate was slower than that recorded for the pure PLGA scaffold. Addition of heparin and BMP2 to the scaffold promoted bone formation at the bone defect in the early stage (Figure 6). The acidic products of some polymers (e.g., polylactic acid) may cause inflammatory reactions and metabolic disorders at the local implantation site. In contrast, the metabolites of CS are neutral or slightly alkaline, which is beneficial to the adhesion, proliferation, and differentiation of cells (Dias et al., 2011). By adjusting the proportions of CS microspheres and calcium phosphate cements, the absorbability can be improved without affecting the overall compressive strength. This composite containing 10% (weight/weight) CS microsphere had a compressive strength of 14.78 ± 0.67 MPa, which is similar to that measured for cancellous bone. In rabbit model of femoral defects, at 24 weeks after implantation, the CS microsphere scaffold had been mostly absorbed and a large number of new bones was observed in the transplantation area (Meng et al., 2015). This is attributed to the fact that CS in the form of microspheres can promote the degradation of calcium phosphate cements and the formation of new bones. In addition, CS microspheres prepared by the emulsion crosslinking method showed better compatibility and osteogenesis with BMSC. Moreover, the degree of bone regeneration *in vivo* was greater than that obtained via the coagulation precipitation method (Xu et al., 2014). Therefore, differences in the preparation of CS microspheres will affect cell expression and bone regeneration. In the treatment of infected defects, appropriate preparation methods should be selected to maximize the utilization of CS microspheres.

### 4.4 Nanofibers

**FIGURE 7:**
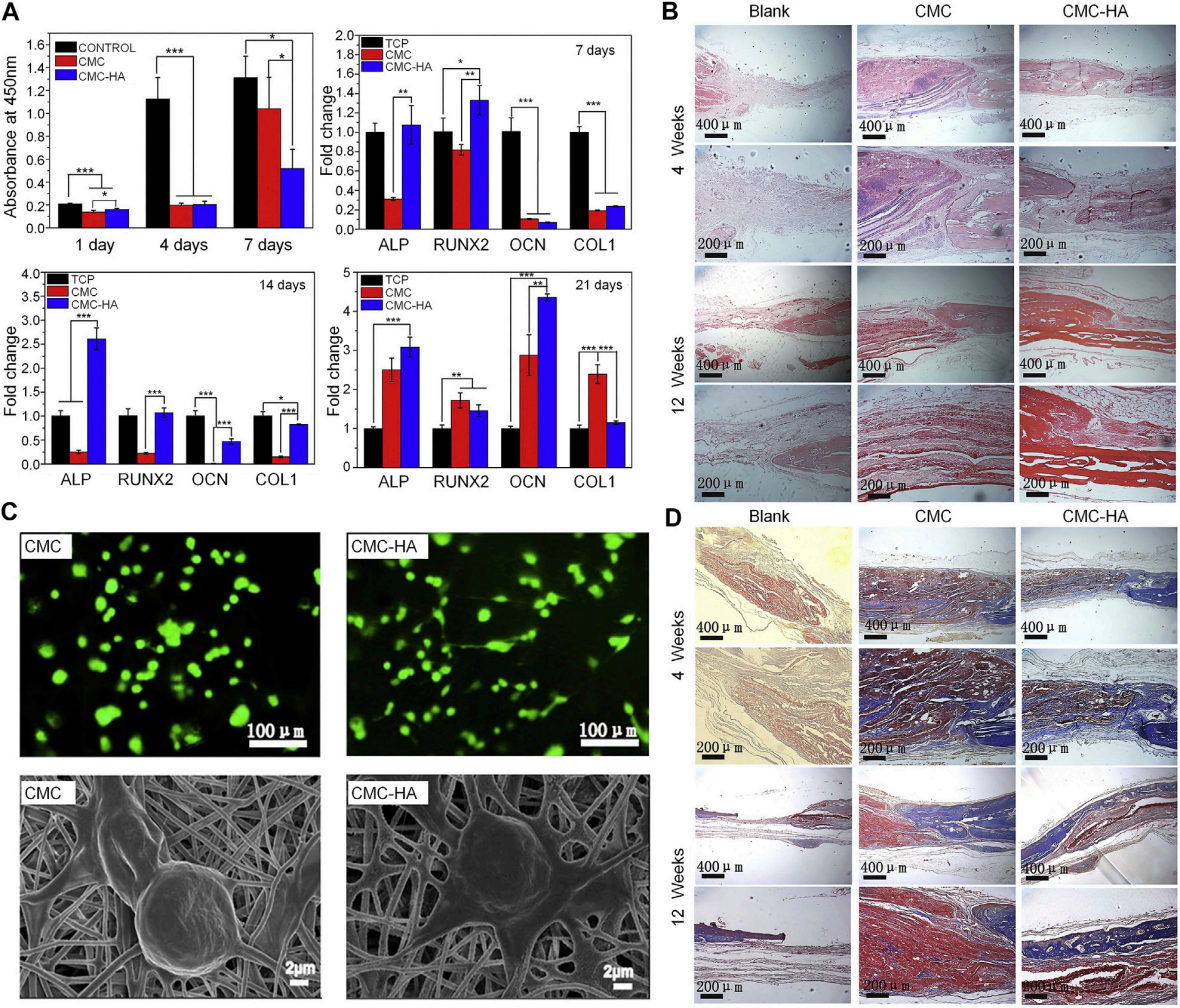
CMC nanofibers have a biomimetic mineralization function *in vitro* and *in vivo* (Zhao et al., 2018). **(A)** CCK-8 test results after 1, 4, and 7 days of culture, and osteogenic gene expression levels analyzed by RT-PCR after 7, 14, and 21 days of culture (*p < 0.05, **p < 0.01, and ***p < 0.001). **(B)** H&E staining results at 4 and 12 weeks. **(C)** Results of LIVE/DEAD staining after 12 h of culture and cell morphology after 6 h of culture using SEM. **(D)** Masson’s trichrome staining results at 4 and 12 weeks. ALP, alkaline phosphatase; CCK-8, Cell Counting Kit-8; CMC, carboxymethyl chitosan; COL1, collagen type 1; HA, hydroxyapatite; H&E, hematoxylin and eosin; OCN, osteocalcin; RT-PCR, reverse transcription-polymerase chain reaction; RUNX2, runt-related transcription factor 2; SEM, scanning electron microscopy.

As a nanofiber scaffold material, its unique characteristics (i.e., large surface area, high porosity, and sufficient mechanical strength) endow CS with extraordinary biological properties (Rasouli et al., 2019). Nanofiber scaffolds can mimic the nanoscale characteristics of the ECM, promote cell adhesion and migration, and enhance metabolism and the transport of nutrients (Sofi et al., 2018). As special biomaterials with nanometer size, CS nanofibers scaffolds can be fabricated by electrospinning, self-assembly, thermal separation, ultrasonic treatment, and chemical synthesis (Ding et al., 2014). Among them, electrospinning is the most commonly used technology for preparing CS nanofibers. By altering the parameters of electrospinning (e.g., voltage, flow rate, viscosity, and solution concentration), the structure and diameter of CS nanofibers can be adjusted to enhance the behavior, function and mechanical properties of cells (Beachley and Wen, 2009). Compared with CS films, CS nanofibers prepared through electrospinning can better promote the adhesion and proliferation of mouse osteoblasts. Moreover, CS nanofibers can stimulate the proliferation and maturation of osteoblasts by inducing the runt-related transcription factor 2 (RUNX2)-mediated regulation of OPN, OCN, and ALP gene expression in osteoblasts through the BMP signaling pathway (Ho et al., 2014). In a model of femoral defects, implantation of the CS nanofiber scaffold promoted bone healing by stimulating and improving the quantity and quality of trabecular bone formation (Ho et al., 2015). CS nanofibers can also improve the mechanical properties of composite biomaterial scaffolds. The fabrication of high-strength nanofiber scaffolds is a major focus in the field of bone defect therapy. The excellent mechanical properties of such scaffolds contribute to maintaining the structural stability of biomaterials *in vivo*. CS lacks sufficient mechanical properties; hence, the mechanical strength of nanofibers can be optimized by combining CS with other materials. CS and HA nanofiber scaffolds crosslinked using genipin can simulate the Young’s modulus of the periosteum, reaching a strength of 142 ± 13 MPa; this strength increases in parallel with the increase in the concentration of HA. By simulating the modulus of bone, this composite scaffold can also enhance the differentiation ability of osteoblast precursor cells and ECM deposition. Therefore, this type of scaffold can be used as a biological template for the formation of new bone (Frohbergh et al., 2012). A novel type of PRP-incorporated electrospun polyvinyl-alcohol-CS-HA nanofibers exhibited remarkable biological and mechanical properties, similar to those of human tissue. Furthermore, this composite scaffold improved the ability of osteoblasts for adhesion and proliferation (Abazari et al., 2019). In addition to physical properties, such as mechanical properties and porosity, composite nanofibers containing CS may offer antibacterial activity. The use of the copper (I)—catalyzed azide-alkyne cycloaddition (CuAAC) reaction to graft polycaprolactone (PCL) to the CS by the electrospinning method has been reported. Subsequently, magnesium-doped hydroxyapatite (Mg-HA) was added to the blend composite to prepare a CS-g-PCL/Mg-HA nanofiber scaffold. The triazole group produced by the CuAAC reaction can interact with lipids on the microbial cell membrane to produce antibacterial properties and enhance the osteoblast activity of MG-63 cells (Sedghi et al., 2020). Although electrospinning is one of the most commonly used methods in bone tissue engineering, it is associated with some challenges. For example, the selection of solvents will affect the cytotoxicity of CS nanofibers, and the process of electrospinning is relatively complicated (Chahal et al., 2019). The preparation of polycationic CS and polyanionic ulvan nanofibers by the molecular self-assembly method can also promote the proliferation of osteoblasts and maintain the morphology of osteoblasts. The manufacturing method is simpler than that of the electrospinning method (Toskas et al., 2012). The side chain groups of CS can also be modified to prepare nanofibers. Compared with the solvent used for electrospinning CS nanofibers, CMC nanofibers are water-soluble, non-toxic, and do not require the removal of acid salts generated during electrospinning (Su et al., 2016). The CMC nanofibers with HA can be prepared by the electrospinning method through simple biomimetic mineralization. CMC nanofibers have more mineral deposits than CS nanofibers at 16 h after mineralization. This observation is mainly attributed to the fact that carboxymethyl groups provide more nucleation sites, which is consistent with the findings described above. CMC nanofibers can effectively promote the differentiation of mouse BMSC *in vitro* and augment osteogenesis in rat calvarial bone defects *in vivo* (Figure 7) (Zhao et al., 2018).

## 5 Conclusion and Prospects

Infected bone defects continue to pose a challenge in the field of orthopedics. CS is a bioactive material commonly used in bone tissue engineering. CS kills bacteria through the combination of positive and negative charge. Moreover, CS promotes the proliferation of osteoblasts by increasing the expression of genes related to calcium binding and mineralization, such as ALP, OPN, and OCN. Also, combination with inorganic and organic molecules (e.g., metal ions, graphene oxide, and nano-HA) improves the osteogenic performance of CS. In addition, these properties of CS can be enhanced by side chain modification, yielding QCS, CMC, SCS, and PCS. Among them, QCS is the most common form; it is characterized by enhanced antibacterial activity, an optimal degree of substitution of 20%, and a critical degree of substitution of 90%. In addition, carboxylation, sulfation, and phosphorylation also promote the antibacterial and osteogenic properties of CS. At present, CS can be applied to the treatment of infected defects in many forms, including hydrogels, coatings, microspheres, and nanofibers, all of which have achieved good therapeutic effects.

Although there have been significant advances in the research on the treatment of infectious bone defects with CS, there are still some deficiencies that need to be implemented to promote its extensive clinical application. First of all, the hydrophobicity of CS greatly limits its application, and the antibacterial property of pure CS is not as effective as that of antibiotics. Although some modification of CS can solve this problem, the modified CS will inevitably produce some toxicity. Furthermore, the synthesis of modified CS is complicated, and it is not easy to control the quantification. Therefore, future research should focus on the development of cell-compatible solvents and modification methods to enhance the antibacterial activity and bone-promoting ability of CS while maintaining good biocompatibility. In addition, the insufficient mechanical properties of CS limit its wide application. The combination of CS with other materials such as inorganic materials can make up for the deficiencies. Therefore, these mixed materials deserve further study. Moreover, there is limited research on the relationship between the degradation rate and MW of CS *in vivo*. Many studies on CS are still in the laboratory stage, and further research is needed to be used as bone graft biomaterial for treating infection in clinical treatment. In summary, future research should focus on the efficiency of CS to maximize its antibacterial and osteogenic properties under physiological conditions and more natural bioactive materials mixed with CS need to be developed to further improve biological performance. Such evidence would help overcome the existing difficulties and provide a new perspective for the treatment of infected bone defects.
